# Are accelerometer-measured sitting and physical activity times associated with muscle mass and strength in healthy young adults in the UAE?

**DOI:** 10.1016/j.heliyon.2024.e30899

**Published:** 2024-05-08

**Authors:** Raneen Mohammed Qadah, Alham Al-Sharman, Reime Jamal Shalash, Ashokan Arumugam

**Affiliations:** a Department of Physiotherapy, College of Health Sciences, University of Sharjah, P.O. Box: 27272, Sharjah, United Arab Emirates; b Neuromusculoskeletal Rehabilitation Research Group, RIMHS–Research Institute of Medical and Health Sciences, University of Sharjah, Sharjah, United Arab Emirates; c Department of Rehabilitation Sciences, Faculty of Applied Medical Sciences, Jordan University of Science and Technology, Irbid, Jordan; d Health Promotion Research Group -Research Institute for Medical and Health Sciences, University of Sharjah, Sharjah, United Arab Emirates; e Sustainable Engineering Asset Management Research Group, RISE-Research Institute of Science and Engineering, University of Sharjah, Sharjah, United Arab Emirates; f Adjunct Faculty, Manipal College of Health Professions, Manipal Academy of Higher Education, Manipal, Karnataka, India

**Keywords:** Physical activity, Accelerometer, Sedentary behavior, Muscle strength, Muscle mass

## Abstract

**Background:**

A high prevalence of obesity, sedentary behavior, and physical inactivity could affect muscle mass and strength in young adults in the United Arab Emirates (UAE). Therefore, we investigated the association of sex, body mass index (BMI), and accelerometer-measured sitting and physical activity (PA) times with skeletal muscle mass index (SMI), hand grip, and thigh muscle strength in healthy young adults in the UAE.

**Methods:**

In this cross-sectional study, 156 healthy young adults (age 21.68 ± 3.01 years, BMI 25.40 ± 4.79 kg/m^2^, 52.6 % women) were included. BMI and muscle mass were recorded using a bioelectrical impedance analyzer. Maximum hand grip strength and thigh muscle torque were assessed using the Jamar-smart hand-dynamometer and Biodex System-4-Pro, respectively. Participants wore a triaxial Fibion accelerometer on their anterior thigh for >10 h per day for 4–7 days to measure their sitting and PA times. Multiple linear regression analyses were used.

**Results:**

Participants spent most of their time sitting (11.37 ± 1.10 h), followed by standing (2.92 ± 0.86 h), walking (1.58 ± 0.55 h), and vigorous intensity PA (4.79 ± 5.85 min) per 16-h day. Sex (p < 0.001) and BMI (p < 0.001) were negatively associated with all muscle mass and strength variables. Men had more muscle mass and strength than women. As BMI increased, muscle mass and muscle strength decreased. Accelerometer-measured sitting and walking times were negatively associated with concentric hamstrings (p = 0.044) and quadriceps torques (p = 0.031), respectively.

**Conclusion:**

Sex, BMI, and accelerometer-measured sitting and walking times were associated with muscle mass and/or muscle strength in healthy young adults. Women and those with a high BMI need interventions to improve their muscle mass and strength. The paradox regarding the association of PA with muscle mass and strength in younger adults may be due to possible influences from other factors (e.g., resistance training, dietary intakes, etc.) superseding that of accelerometer-measured PA.

## 1 Introduction

In the Middle East, there is a lack of physical activity (PA) engagement, and this has made sedentary behavior a main cause of public health problems [1]. Adult Emiratis and expatriates (89 % of the United Arab Emirates [UAE] population) have high rates of obesity, diabetes mellitus, and cardiovascular disease due to, but not limited to, acculturation and the rapid socioeconomic transition that the country went through [2,3]. A cross-sectional study investigating (waist-worn) accelerometer-measured sedentary behavior and PA in young adults in the UAE revealed that they spend about 80 % of their waking hours in sedentary activities [1]. Hence, the low prevalence of PA among the UAE population is a major concern because it is an independent risk factor for many noncommunicable diseases [4,5]. PA and sedentary behaviour are the main factors known to influence the metabolic processes involving body composition [6]. PA reduces body fat mass, increases muscle mass and strength, and improves physical function [6]. Engaging in regular daily PA helps in preventing sarcopenia [7].

Sarcopenia is a muscle disease characterized by a decline in the skeletal muscle mass and strength [8]. Although it is primarily reported in older adults, sarcopenia can affect people of all ages, including young adults [9]. Sarcopenia has been noted in individuals of a younger age, including those affected by inflammatory conditions, physical limitations, nutritional deficiencies, and cachexia [10]. Insufficient muscular strength, irrespective of age, increases the likelihood of facing functional limitations, poor physical performance, injuries related to activities, and negative health consequences [11]. A certain level of muscle strength is required for the execution of activities like squatting, hopping, jumping, kicking, throwing, and sprinting [11,12]. Young individuals with inadequate muscular strength are less likely to engage in daily PA, thereby making them more susceptible to disease risk factors and compromised metabolic health [11,12].

The risk of developing sarcopenia in early adulthood, resulting in exaggerated muscle dysfunction in later life, is still in the early stages of exploration despite its clinical significance [13]. While the prevalence of sarcopenia among young individuals is comparatively lower than in older adults, the extended duration of sarcopenia during youth may lead to more significant long-term health issues [13]. Consequently, it is crucial to identify and treat sarcopenia early in young individuals for primary prevention and to limit its progression [13]. Estimating the global sarcopenia prevalence in early adulthood is challenging, given the limited epidemiological research on this condition [13].

Two systematic reviews concluded that PA is effective in preventing sarcopenia [14,15]. A recent cross-sectional study conducted on Arab men (aged 47.7 ± 15.4 years) found an association between time spent in recreational moderate-to-vigorous intensity PA and lean muscle mass [16]. In addition, men have been reported to have more skeletal muscle compared to women [17]. An important factor that may be associated with a decrease in muscle mass is obesity [18], which is more prevalent among women than men and increases as a person ages [19]. Obesity affects all the body's physiological functions, and it has health threats [19]. For instance, it increases the risk of developing metabolic diseases (e.g., type 2 diabetes mellitus), musculoskeletal diseases (e.g., osteoarthritis), Alzheimer's disease, cardiovascular diseases (e.g., hypertension and stroke), depression, and some cancer types (affecting breast, kidney, and liver) [19].

The measurement of the muscle mass amount is the basis for assessing sarcopenia [20]. Muscle mass can be measured using a variety of methods, including computed tomography, magnetic resonance imaging, dual-energy X-ray absorptiometry (DXA), and bioelectrical impedance analysis (BIA) [20]. Among them, DXA and BIA are the most used methods, as they are noninvasive [20]. Some disadvantages of DXA include that it is expensive, hard to transport from one place to another, involves (minimal) X-ray exposure, and a lack of availability at all clinics [21]. On the other hand, BIA is less expensive, less harmful, more accessible, easy to use, has high reproducibility, and has a high speed of information processing [20]. It is a valid tool for assessing the body composition (muscle mass, total body water, extracellular water, intracellular water, fat mass, fat-free mass, etc.) and it has shown a good correlation with DXA [21,22].

Muscle strength is one of the important components of physical fitness [23]. Maximum muscle strength is typically achieved between 20 and 30 years and starts to decline around the age 40 years [24]. A study on healthy men and women aged 20–94 years found that muscle volume and functional parameters are closely related with age and PA in men but not in women [25]. Also, it has been reported that handgrip strength is associated with body mass index (BMI) [26]. Keevil et al. found that a high body mass index (BMI) is associated with a high grip strength, but a high waist circumference is associated with a low hand grip strength [27]. Furthermore, a positive correlation between BMI and isokinetic muscle strength of the quadriceps, triceps, and abdominal muscles has been reported in children [28].

A systematic review reported that objective measures (accelerometers, pedometry etc.) of PA are moderately associated with lower limb muscle size and muscle strength in the general population [29]. In addition, Leblanc et al. reported weak associations (r = 0.139–0.186) between self-reported PA and upper body strength, and accelerometer-measured PA and lower body strength. They found age, sex, and BMI contribute to muscle strength variability in healthy adults (aged 20–91 years) [30].

Most of the existing studies have explored the associations of sex, BMI, sedentary behavior, and PA levels with muscle mass and muscle strength in older adults [[31], [32], [33], [34], [35]]. These associations should be studied on young adults as well, because there is a high prevalence of obesity, an increase in sedentary behaviour, a decrease in PA, and a risk of sarcopenia in young adults which could affect their muscle mass and strength [[36], [37], [38]]. To the best of our knowledge, no study has yet investigated the association of thigh-worn accelerometer-measured sitting and PA times with SMI, hand grip, and thigh muscle strength in young adults in the UAE. Therefore, the aim of this study was to investigate the association of sex, BMI, and (thigh-worn) accelerometer-measured sedentary (sitting) and PA times with skeletal muscle mass index (SMI), and maximum hand grip and (isometric and isokinetic) thigh muscle strength in healthy young adults of the UAE. We hypothesized that there will be an association of sex, BMI, and accelerometer-measured sitting and PA times with SMI, and hand grip and thigh muscle strength.

If there is an association between sitting or PA times and muscle mass and strength, then young adults could focus on engaging in PA to increase or maintain their optimal muscle mass and strength. Furthermore, if there are BMI and sex differences in these associations, future longitudinal cohort studies could explore the predictive association of these factors with muscle mass and strength in young adults in the UAE. The current study may inform the need for randomized clinical trials investigating the effectiveness of interventions targeting maintaining a healthy BMI, reducing sedentary behavior, and increasing PA on muscle mass and strength in young adults of both sexes in the UAE.

## 2 Methods

### 2.1 Study design and setting

A cross-sectional study was conducted at the University of Sharjah, United Arab Emirates. Ethical approval was obtained from the Research Ethics Committee of University of Sharjah (REC-23-01-18-02-S).

### 2.2 Sample size

The G*Power 3.1.9.7 software was used for sample size computation. For an effect size of 0.08, an alpha value of 0.05, a power of 0.80, and four independent (predictor) variables to be included in multiple linear regression, the minimum sample size required is 155.

### 2.3 Study participants

A convenience sample of healthy young adults aged between 18 and 35 years was recruited through adverts on social networking websites (e.g., Twitter, LinkedIn, and Facebook), mobile applications (e.g., WhatsApp and Botim), university notice boards, flyers, and/or word of mouth. One hundred seventy-three young adults volunteered to participate in the study. Participants were excluded from the study: if they had a BMI <18 kg/m^2^, were pregnant or lactating mothers, had any metal implants or a pacemaker, had a self-reported history of musculoskeletal pain in the past 6 months or clinically diagnosed disorders, suffered from any chronic disease that would interfere with dietary intake, suffered from psychological diseases, chronic diseases, asthma, cardiovascular diseases, cancer, organ failure, uncontrolled metabolic diseases (diabetes, anemia or thyroid disease), electrolyte imbalance, musculoskeletal problems, systemic diseases or recent surgeries that might impact PA levels, had any physical impairments (amputation, paralysis, etc.) or were on medications that might interfere with body composition such as corticosteroids, hormones, and endocrine diseases or autoimmune diseases medications, and if they were hospitalized within the past three months for any illness.

### 2.4 Instrumentation

The following devices were used in the study: the Fibion (triaxial) accelerometer devices (Fibion Inc, Jyväskylä, Finland); the MC-780PMA Multi Freq Body Composition Analyzer (Tanita MC-780MA, Japan); the Jamar Smart hand dynamometer (Performance Health Supply, Cedarburg, USA); and the Biodex system 4 pro and MVP (Biodex Medical System, Inc, Shirley, United states of America).

### 2.5 Procedure

Prior to data collection, an information sheet and a screening questionnaire were given to all volunteers and their questions were answered clearly before obtaining their informed consent. They filled the extended version of the Nordic Musculoskeletal questionnaire (NMQ-E) for ruling out any history of musculoskeletal symptoms (in the previous 12 months and the past seven days) [39]. All eligible participants filled a socio-demographic information sheet that included age, sex, nationality, occupation, level of education, marital status, medical history, surgical history, medications, etc. A qualified physiotherapist (RMQ) collected anthropometric and accelerometer data.

#### 2.5.1 Anthropometric measurements

The height of participants was measured using a stadiometer. The skeletal muscle mass of each segment (kg), weight, and BMI were assessed using the MC-780PMA Multi Freq Body Composition Analyzer (Tanita MC-780MA, Japan). The participants were instructed to remove their shoes, socks, and any metal objects before standing on the BIA platform at the time of measurement during the day.

A pilot study of 10 participants was conducted to assess the reliability of this particular BIA device. The BIA measurements were assessed 3 times (before food, 30 min after food, and after one week before food). All the variables of interest were found to have an excellent reliability: weight (ICC: 1.000), BMI (ICC: 0.937), muscle mass of the right and left upper limbs (ICC: 0.989, 0.995), muscle mass of the right and left lower limbs (ICC: 0.999, 0.996), appendicular skeletal muscle mass (ASM) (ICC: 0.999), and SMI (ICC: 0.990) This device has been found to show a moderate correlation between the relevant parameters (particularly the SMI - r = 0.614) and computed tomography-measured SMI at the third lumbar vertebra [40].

As strict adherence to fasting is not necessary when measuring fat mass in clinical settings using BIA, our participants were not asked to observe fasting before BIA measurements [[41], [42], [43]]. The differences in fat mass found before and after consuming water/food or engaging in PA are small and clinically insignificant [[41], [42], [43]]. Before each assessment, all the BIA device accessories (e.g., sensors, electrodes, etc.) were checked for wear or damage, and the scale was calibrated by setting it at “zero”. The process of BIA measurements were adopted from Merrigan et al. [44]. After the BIA device had completed analyzing the data, the skeletal muscle mass of each segment (kg) was used to estimate the ASM, which is the sum of the muscle mass of all four limbs [20]. The SMI was derived by dividing the ASM (kg) by body weight (kg) and multiplying by 100 [17].

#### 2.5.2 Accelerometer–measured sedentary behavior and PA

The sedentary behaviour and PA levels were assessed using a triaxial Fbion accelerometer (Fibion Inc, Jyväskylä, Finland). The Fibion has an overall accuracy of 85–89 %, with a high accuracy in detecting lying, sitting, and standing [45]. It has demonstrated a good to excellent validity in measuring sitting time (ICC: 0.87), standing time (ICC: 0.84), and walking time (ICC: 0.97) when compared to the thigh-worn ActivPAL4 [46]. This accelerometer has been used in several studies for measuring sitting and different PA types in the UAE [[46], [47], [48], [49], [50]]. The participants wore the device on the proximal third of the anterior thigh for four to seven days (with a minimum of three weekdays and one weekend day) for at least 10 h per day, using a non-allergic adhesive tape (Hypafix®) [46,[48], [49], [50], [51], [52], [53]]. They removed it when sleeping, showering or swimming.

Fibion data were obtained and processed from CSV files that contained minute-by-minute and day-by-day data [[48], [49], [50],^52^,^54^]. Night-time data were excluded using each participant's daily diary log. Then data were normalized to 16 h using this formula: normalized sitting/activity time = (Fibion-recorded sitting/activity time)/(Fibion-recorded wear time)*16 h; here 16 h refer to the maximum wake time per day [55,56]. Sitting (sedentary behavior), standing, walking and vigorous PA times were included in the analysis. Some of these functional activities were included in previous studies but vigorous activity was not included in any of them [47,48,57,58].

#### 2.5.3 Isometric hand grip strength

Maximal isometric grip strength of the dominant hand was assessed using the Smart Jamar hand dynamometer (Performance Health Supply, Cedarburg, USA). The Jamar hand dynamometer device is considered the gold standard instrument for measuring the maximal isometric hand grip strength as recommended by the American Society of Hand Therapists [59,60]. Hand grip strength testing with this device has demonstrated a high test-retest reliability [[61], [62], [63]]. Hand dominance was assessed using the Edinburgh handedness inventory – short form [64].

Before each assessment started, the therapist made sure that the reading on the dynamometer dial was “0” kg. Each participant sat on a chair (back supported) with their arm rested on an arm rest, the shoulder adducted, the elbow flexed to 90°, the forearm in neutral position, and the wrist in 30° extension and 15° of ulnar deviation, and then squeezed the dynamometer (on the second handle position) with a maximal effort for 3 s [60,65,66] They performed three trials with a 1 min break between each of them and then the average was calculated [30,67]. The Isometric_HG_ was recorded in Kg, converted into Newtons (by multiplying with 9.81 m/s^2^) [68] and allometrically scaled using the equation suggested by Jaric et al. (N/(body weight (kg))^0.67^ [69].

#### 2.5.4 Thigh muscle strength

Thigh muscle strength was assessed using the Biodex system 4 pro (Biodex Medical System, Inc, Shirley, United states of America). The participants warmed up before the test by performing 5 min of self-paced static cycling, two submaximal knee extension contractions at 10 %, two contractions at 50 %, and two contractions at 90 % effort [30,70,71]. The self-preferred dominant leg, used to kick a ball, was tested [72,73]. Consistent verbal encouragement and visual feedback on the computer monitor were given to the participant by the assessor [74]. The therapist made sure that the device was calibrated before each participant. The Biodex software automatically calibrates the device once it is switched on. The effect of gravity on the leg was corrected by placing the knee joint at an angle of 35–40° of extension for all the participants [75].

##### 2.5.4.1 Isometric thigh muscle strength

The participants performed three isometric contractions at a knee angle of 60° [74]. The participants started the test in a seated position by pushing the lever in the extension direction and repeated the test by pulling the lever in flexion direction as hard as they could. Each contraction was held for 5 s followed by a 30 s rest between trials [76]. They then rested for 5 min [30]. Isometric peak torque normalized to body weight (Nm/kg) for quadriceps (Isometric_Q_) and hamstring (Isometirc_H_) were calculated. The value was multiplied by 100 to obtain a functional test score.

##### 2.5.4.2 Isokinetic thigh muscle strength

The participants performed five reciprocal concentric quadriceps and hamstring contractions at 60°/s (within a 90° range of motion) as hard and as fast as they could, followed by 5 min of rest [30,77]. Therapist's verbal encouragement and visual feedback via the Biodex monitor were provided during the test. The isokinetic peak torque (normalized to body weight – Nm/kg) for the quadriceps (Con_Q_) and hamstring (Con_H_) were calculated. The torque values were multiplied by 100 to get functional test scores.

### 2.6 Statistical analysis

Data normality was checked using the Kolmogorov-Smirnov test and histograms. Descriptive statistics of data were presented as mean and standard deviation (SD), or frequencies (with %). Multiple linear regression analyses were used to analyze the association between independent variables (sex; BMI; sitting, standing, walking, and vigorous PA times per 16-h day) and dependent variables (SMI; IsometricHG; IsometricH, and IsometricQ; ConH and ConQ). All statistical analysis were performed using IBM SPSS Statistics version 28. A p value of <0.05.

The degree of multicollinearity of predictor variables was measured using the variance inflation factor (VIF), and any variable with a VIF more than 3.0 was eliminated from the model. Standing time was excluded from the analysis because it inflated the VIF of other independent variables. The box and whisker plots were used to retain mild values (between 1.5 and 3 interquartile range (IQR) reflecting normal population variability) and exclude extreme outliers (>3 times the IQR length) [78]. Independent t tests were used to compare sedentary and PA times between men and women. The effect size of between-sex differences was assessed using Cohen's d, which was interpreted as small (0.20–0.50), medium (0.51–0.80), or large (>0.80) [79].

## 3 Results

One hundred and fifty-six healthy young adults (mean age of 21.68 (±3.01); 47.4 % men) were included in the analysis. Those did not meet the eligibility criteria (n = 1), withdrew from the study (n = 3), had invalid accelerometer-measured PA (n = 3), lost the accelerometer device (n = 7) or extreme outliers (n = 3) were excluded. Fifty percent had a BMI <25 kg/m^2^, while the rest were >25 kg/m^2^. Participants studying or having a bachelor's degree accounted for 87.8 %, while others were master students. Most participants were Arab individuals (92.9 %), while the others were Asians (4.5 %) or Africans (2.6 %). Table 1 summarizes socio-demographic and anthropometric characteristics of the participants.

**Table 1:** Socio-demographic and anthropometric characteristics of participants (n = 156).

Variable	Value
Sex	
*Women*	82 (52.6 %)
*Men*	74 (47.4 %)
BMI (kg/m^2^)	
*> 25*	78 (50 %)
*< 25*	78 (50 %)
Educational level	
*Bachelor's degree*	137 (87.8 %)
*Master's degree*	19 (12.2 %)
Nationality	
*Arabs*	139 (89.1 %)
*Emiratis*	6 (3.8 %)
*Asians*	7 (0.7 %)
*Africans*	4 (2.6 %)
Age (years)	21.68 ± 3^a^
Weight (kg)	72.62 ± 17.71^a^
BMI (kg/m^2^)	25.40 ± 4.79^a^
Height (cm)	168.57 ± 9.47^a^

a Mean ± standard deviation.

Participants spent most of their time in sitting (11.37 ± 1.10 h), followed by standing (2.92 ± 0.86 h), and walking (1.58 ± 0.55 h). They spent very less time in vigorous intensity PA (4.79 ± 5.85 min). Sitting (mean difference [95 % CI] = 8.80 [−12.18, 29.78] minutes; P = 0.410) and walking (5.26 [−6.18, 14.60] minutes; P = 0.431) times were not significantly different between men and women, but standing (−17.88 [−34.12, −1.65]; P = 0.032; effect size, Cohen's d = 0.349 [small]) and vigorous PA (4.93 [3.24, 6.61]; P < 0.001; Cohen's d = 0.926 [large]) times were significantly different between them. Table 2 summarizes the accelerometer-measured sitting and physical activity variables, anthropometric measures related to muscle mass, and muscle strength of all participants.

**Table 2:** Accelerometer-measured sitting and physical activity variables, anthropometric measures related to muscle mass, and muscle strength of all participants.

Variable	Total (n = 156)	Women (n = 82)	Men (n = 74)
Sitting (min/16-h day)	681.90 ± 66.17	677.73 ± 65.02	686.52 ± 67.56
Standing (min/16-h day)	175.06 ± 51.87	183.25 ± 66.25	156 ± 54.64
Walking (min/16-h day)	94.96 ± 32.77	92.96 ± 28.22	97.17 ± 37.26
Vigorous (min/16-h day)	4.79 ± 5.85	1.12 ± 2.86	5.93 ± 10.95
Muscle mass of the right upper limb (kg)	2.59 ± 0.98	1.84 ± 0.46	3.42 ± 0.68
Muscle mass of the left upper limb (kg)	2.56 ± 0.96	1.83 ± 0.45	3.38 ± 0.67
Muscle mass of the right lower limb (kg)	8.25 ± 2.25	6.60 ± 1.13	10.10 ± 1.68
Muscle mass of the left lower limb (kg)	8.19 ± 2.21	6.57 ± 1.16	9.98 ± 1.65
SMI (%)	29.6 ± 4.51	26.33 ± 2.32	33.462 ± 3.25
Isometric_HG_ (Nm/kg*100)	17.43 ± 5.73	14.23 ± 3.40	20.98 ± 4.91
Con_H_ (Nm/kg*100)	92.63 ± 34.94	70.93 ± 23.4	116.66 ± 28.54
Con_Q_ (Nm/kg*100)	191.17 ± 63.82	151.30 ± 43.25	235.34 ± 53.00
Isometirc_H_ (Nm/kg*100)	103.03 ± 40.14	81.13 ± 28.62	127.30 ± 37.10
Isometric_Q_ (Nm/kg*100)	189.85 ± 61.49	158.29 ± 43.56	224.83 ± 59.69

Values are presented as mean ± standard deviation.

Con_H_ = isokinetic hamstring strength, Con_Q_ = isokinetic quadriceps strength, Isometirc_H_ = isometric hamstring strength, Isometric_Q_ = isometric quadriceps strength, Isometric_HG_ = maximum isometric grip strength, SMI=Skeletal muscle mass index.

### 3.1 SMI

Sex and BMI (<0.001) were significantly associated with SMI (p < 0.001; Table 3). SMI was lower by 7.73 kg/m^2^ in women than in men. Every 1 kg/m^2^ increase in BMI was associated with a 0.36 kg/m^2^ decrease in SMI. Sitting time and PA times were not significantly associated with SMI.

**Table 3:** Variables associated with skeletal muscle mass index.

Variable	β Coefficient	95 % CI	P-value	_Adjusted R_^2^
Constant	46.9	(40.81, 52.98)	<0.001	76.2 %
Sex	−7.73	(-8.51, −6.96)	**<0.001**	
BMI (kg/m^2^)	−0.36	(-0.43, −0.28)	**<0.001**	
Sitting (min/16-h day)	−0.01	(-0.01, 0.00)	0.163	
Walking (min/16-h day)	−0.01	(-0.02, 0.01)	0.530	
Vigorous (min/16-h day)	−0.03	(-0.10, 0.04)	0.431	

BMI = body mass index.

### 3.2 Isometric_HG_

Sex and BMI were significantly associated with Isometric_HG_ (p < 0.001; Table 4). Isometric_HG_ was lower by 7.03 N/kg in women than in men. Every 1 kg/m^2^ increase in BMI was associated with a 0.39 N/kg decrease in Isometric_HG_. Sitting time and PA times were not significantly associated with Isometric_HG_.

**Table 4:** Variables associated with normalized isometric hand grip srength.

Variable	β Coefficient	95 % CI	P-value	_Adjusted R_^2^
Constant	37.04	(26.65, 47.44)	<0.001	51.0 %
Sex	−7.03	(-8.36, −5.70)	**<0.001**	
BMI (kg/m^2^)	−0.39	(-0.51, −0.26)	**<0.001**	
Sitting (min/16-h day)	−0.01	(-0.02, 0.01)	0.248	
Walking (min/16-h day)	−0.02	(-0.04, 0.01)	0.219	
Vigorous (min/16-h day)	0.07	(-0.05, 0.10)	0.244	

BMI = body mass index.

### 3.3 Isometirc_Q_ torque

Sex and BMI were significantly associated with Isometirc_Q_ torque (p < 0.001; Table 5). Isometirc_Q_ torque was lower by 67.78 Nm/kg in women than in men. A 1 kg/m^2^ increase in BMI was associated with a 3.44 Nm/kg decrease in Isometric_Q_ torque. Sitting time and PA times were not significantly associated with Isometirc_Q_ torque.

**Table 5:** Variables associated with normalized isometric quadriceps torque.

Variable	β Coefficient	95 % CI	P-value	Adjusted _R_^2^
Constant	368.52	(232.00, 505.04)	<0.001	35.5 %
Sex	−67.78	(-85.23, −50.33)	**<0.001**	
BMI (kg/m^2^)	−3.44	(-5.12, −1.77)	**<0.001**	
Sitting (min/16-h day)	−0.07	(-0.23, 0.09)	0.406	
Walking (min/16-h day)	−0.14	(-0.47, 0.20)	0.414	
Vigorous (min/16-h day)	0.87	(-0.65, 2.40)	0.261	

BMI = body mass index.

### 3.4 Isometirc_H_ torque

Sex and BMI were significantly associated with with Isometirc_H_ torque (p < 0.001; Table 6). Isometirc_H_ was lower by 48.66 Nm/kg in women than in men (Table 6). Every 1 kg/m^2^ increase in BMI was associated with a 2.12 Nm/kg decrease in Isometirc_H_ torque. Sitting time and PA times were not significantly associated with Isometirc_H_ torque.

**Table 6:** Variables associated with normalized isometric hamstrings torque.

Variable	β Coefficient	95 % CI	P-value	Adjusted _R_^2^
Constant	198.57	(110.94, 286.19)	<0.001	37.6 %
Sex	−48.66	(-59.86, −37.46)	**<0.001**	
BMI (kg/m^2^)	−2.12	(-3.19 -1.04)	**<0.001**	
Sitting (min/16-h day)	−0.12	(-0.12, 0.09)	0.820	
Walking (min/16-h day)	−0.09	(-0.30, 0.12)	0.404	
Vigorous (min/16-h day)	0.13	(-0.84, 1.11)	0.787	

BMI = body mass index.

### 3.5 Con_Q_ torque

Sex and BMI were significantly associated with with Con_Q_ torque (p < 0.001; Table 7).

**Table 7:** Variables associated with normalized isokinetic concentric quadriceps torque.

Variable	β Coefficient	95 % CI	P-value	Adjusted _R_^2^
Constant	434.61	(315.45, 553.78)	<0.001	54.4 %
Sex	−88.02	(-103.26, −72.79)	**<0.001**	
BMI (kg/m^2^)	−4.42	(-5.88, −2.96)	**<0.001**	
Sitting (min/16-h day)	−0.09	(-0.23, 0.06)	0.237	
Walking (min/16-h day)	−0.32	(-0.61, −0.03)	**0.031**	
Vigorous (min/16-h day)	0.75	(-0.58, 2.08)	0.268	

BMI = body mass index.

Con_Q_ torque was lower by 88.02 Nm/kg in women than in men (Table 7). Con_Q_ torque was negatively associated with walking time (p = 0.031). Every 1 min increase in walking time was associated with a 0.32 Nm/kg decrease in Con_Q_ torque. An 1 kg/m^2^ increase in BMI was associated with a 4.42 Nm/kg decrease in Con_Q_ torque. Sitting and other PA times were not significantly associated with Con_Q_ torque.

### 3.6 Con_H_ torque

Sex and BMI were significantly associated with Con_H_ torque (p < 0.001; Table 8). Con_H_ torque was lower by 46.98 Nm/kg in women than in men. A 1 kg/m^2^ increase in BMI was associated with a 1.74 Nm/kg decrease in Con_H_ torque. Con_H_ torque was negatively associated with sitting time (p = 0.044). Every 1 min in sitting time was associated with a 0.08 Nm/kg decrease in Con_H_ torque. PA times were not significantly associated with Con_H_ torque.

**Table 8:** Variables associated with normalized isokinetic concentric hamstrings torque.

Variable	β Coefficient	95 % CI	P-value	Adjusted _R_^2^
Constant	230.10	(162.91, 297.28)	<0.001	50.5 %
Sex	−46.98	(-55.57, −38.39)	**<0.001**	
BMI (kg/m^2^)	−1.74	(-2.56, −0.92)	**<0.001**	
Sitting (min/16-h day)	−0.08	(-0.16, 0.00)	**0.044**	
Walking (min/16-h day)	−0.16	(-0.32, 0.01)	0.062	
Vigorous (min/16-h day)	0.47	(-0.28, 1.22)	0.216	

BMI = body mass index.

## 4 Discussion

A significant association of sex and BMI was found with all dependent variables including SMI, Isometric_HG_, Isometric_Q_ torque, Isometirc_H_ torque, Con_Q_ torque, and Con_H_ torque. The accelerometer-measured walking and sitting times were negatively associated with Con_Q_ and Con_H_ torques, respectively.

### 4.1 Association of sex with muscle mass and strength

We found that men have higher SMI than women in young adults, this finding is consistent with previous studies conducted on young and middle-aged adults [80,81]. These significant differences between sexes could be due to increased testosterone levels in men, as it is associated with an increase in muscle mass [80]. Moreover, our findings are consistent with previous studies conducted on young and older adults which stated that male participants generally have greater Isometric_HG_ compared to female participants [32,[82], [83], [84], [85]]. Furthermore, we found isokinetic and isometric thigh muscle strength to be greater in male participants than in female participants as evident in several previous studies [[86], [87], [88]]. The larger size and mass of muscles in men would account for their greater muscle strength than in their female couterparts [82].

Our secondary analysis revealed that women stood 18 min longer than men, while men performed 5 min more vigorous PA than women, per day, on average. There were no significant differences in sitting and walking times between men and women. Our results are consistent with a recent study that stated both young men and women spent high amounts in sedentary behaviour, and that men spent significantly greater time in moderate-to-vigorous intensity PA than women in the UAE [89]. Future studies exploring factors associated with between-sex differences of PA levels are required in the UAE.

### 4.2 Association of BMI with muscle mass and strength

Our participants with a higher BMI had a lower SMI, but previous studies had reported an increase in absolute skeletal muscle mass with an increase in body mass [90,91]. Moreover, we found that participants with a higher BMI had decreased Isometric_HG_ and (isokinetic and isometric) thigh muscle torque. On the contrary, previous studies reported a positive association of BMI with Isometric_HG_ (in adults) [26], quadriceps, triceps, and abdominal muscles strength (in children) [28]. However, BMI may not be the most appropriate indicator of obesity because it incorporates, but does not separate, lean mass in its calculation which is a determinant of muscle strength; and it does not indicate the shape of the body. A previous study looked at the association of waist circumference (a clinical sign of central obesity) with Isometric_HG_ and found that they are inversely proportional [27].

### 4.3 Association of accelerometer-measured sitting with muscle mass and strength

Despite our insignificant findings for SMI, previous studies conducted on community-dwelling older adults found that longer sitting time is associated with a lower percentage of lean mass or muscle mass [35,92]. Since sarcopenia commonly affects older adults, each hour of daily sitting has been reported to increase the risk of sarcopenia by 33 % in this population [35,93]. Further studies are warranted to confirm these findings in young adults. However, we found a significant negative association between accelerometer-measured sitting time and Con_H_ torque. A cohort study conducted on middle-aged adults found a robust association between sitting time and Isometric_HG_ [94]. These between-study differences might be due to the type of study design, the age of participants, the type of accelerometers used, and adherence to accelerometer measurement procedures.

### 4.4 Association of accelerometer-measured PA times with muscle mass and strength

None of the PA times were significantly associated with SMI and Isometric_HG_, and no significant associations were detected between vigorous PA and other muscle strength variables. On the contrary, a recent study conducted on Arab men (mean age: 47.7 years) found an association between self-reported moderate-vigorous PA and lean muscle mass [16]. Self-reported measures do not correlate well accelerometer data and they are susceptible to biases related to social desirability [[95], [96], [97]].

We found that accelerometer-measured walking time was negatively associated with Con_Q_ torque. Coversely, a study conducted on healthy adults that reported weak associations (explaining 1–3 % of variance) of PA variables such as total energy expenditure or light intensity activity time with upper or lower body muscle strength [30]. Even so, in the latter study, the age range of participants was wide and not specific to one population; and the study was conducted in multiple sites which might cause inconsistency in data collected [30]. Disagreements regarding the association of PA with muscle mass and muscle strength in younger adults may be due to stronger influences of other factors (e.g., resistance training, dietary intakes, etc.) that outweigh the significance of accelerometer-measured PA. The World Health Organization 2020 PA guidelines recommend that adults perform at least two days of muscle strengthening exercises per week in addition to a minimum requirement of 150–300 min of moderate-intensity PA, 75–150 min of vigorous intensity PA, or an equivalent combination of moderate or vigorous intensity activity distributed during the week for optimal health benefits [98].

Several studies investigated the association of PA with muscle strength and muscle mass, but their instruments, PA variables, or age groups were different than our study [31,50,99]. A systematic review and meta-analysis noted that higher PA and lower sedentary behaviour are associated with greater muscle strength in community-dwelling older adults [99]. However, studies addressing such associations of accelerometer-measured sitting and PA times with intrument-measured SMI, hand grip and thigh muscle strength in young adults are further warranted.

### 4.5 Strengths and limitations of the study

To the best of our knowledge, this is the first cross-sectional study that investigated the association of thigh-worn accelerometer-measured sedentary behavior (sitting) and PA times with muscle mass and strength in healthy young adults of the UAE. We did objective measurements of muscle mass; hand and thigh muscle strength; and sitting and PA times using reliable and valid instruments. In addition, the sample size was calculated a priori, and the sample included almost equal proportion of the sexes (men and women) and BMI (normal and overweight/obese) categories. Future longitudinal cohort studies are needed to explore the predictive association of these factors (sex, BMI, sedentary behaviour and PA levels) with muscle mass and strength in young adults. Although we assessed only maximal isometric hand grip strength for the upper extremity, it is a reliable indicator of upper extremity functional ability [[100], [101], [102]]. Moreover, the BIA measurements were not taken in a specific time of the day but according to our pilot study and other previous studies strict adherence to fasting is not necessary and the difference of the measurements after engaging in PA are small and clinically insignificant; therefore, it is a reliable way of measuring [[41], [42], [43]]. Since individuals with a BMI of <18 kg/m^2^ were excluded, future studies including such individuals are warranted in this area. Also, future randomized clinical trials are recommended to investigate the effectiveness of interventions targeting maintaining a healthy BMI, reducing sedentary behaviour, and increasing PA and strength training on muscle mass and muscle strength in young adults of both sexes.

## 5 Conclusion

We found significant associations for sex and BMI with SMI, Isometric_HG_, Isometric_Q_ torque, Isometirc_H_ torque, Con_Q_ torque, and Con_H_ torque in healthy young adults in the UAE. Men had significantly more muscle mass and muscle strength than women, and as BMI increased, muscle mass and muscle strength decreased. Moreover, the accelerometer-measured walking and sitting times were found to be negatively associated with ConQ and Con_H_ torques respectively. The current study highlights the need for young adults, especially, women and those with a high BMI to focus on interventions to improve their muscle mass and muscle strength. The paradox surrounding the association of PA with SMI and thigh muscle strength in younger adults may be due to the possible impact of other factors (e.g., muscle strength straining, nutritional intakes, etc.) superseding that of accelerometer-measured PA times.

## Ethical approval

This study was approved by the Research Ethics Committee of University of Sharjah (REC-23-01-18-02-S). Informed consent was obtained from all participants for the use of their data for scientific research purposes and publication.

## Funding statement

The accelerometers and hand held dynamometer used in the study were funded by the Office of Vice Chancellor for Research and Graduate Studies, University of Sharjah.

## Data availability statement

The datasets used in the study are available from the corresponding author on reasonable request. The data included in the study has not been deposited previously in a publicly available repository.

## CRediT authorship contribution statement

**Raneen Mohammed Qadah:** Writing – original draft, Visualization, Validation, Project administration, Methodology, Investigation, Formal analysis, Data curation, Conceptualization. **Alham Al-Sharman:** Writing – review & editing, Visualization, Supervision, Methodology, Conceptualization. **Reime Jamal Shalash:** Writing – review & editing, Validation, Investigation, Data curation, Conceptualization. **Ashokan Arumugam:** Writing – review & editing, Visualization, Validation, Supervision, Resources, Project administration, Methodology, Funding acquisition, Formal analysis, Conceptualization.

## Declaration of competing interest

The authors declare that they have no known competing financial interests or personal relationships that could have appeared to influence the work reported in this paper.
