# Supplementary material for: Are accelerometer-measured sitting and physical activity times associated with muscle mass and strength in healthy young adults in the UAE?
Source: Heliyon. 2024 May 8;10(10):e30899. doi: 10.1016/j.heliyon.2024.e30899 (PMC11103532; doi:10.1016/j.heliyon.2024.e30899)
Supplement: Multimedia component 1 [file mmc1.docx]

**Supplementary materials:**

**Participants sociodemographic information and screening form**

- Name/initials (optional):
- Age:
- Sex:

□ male □ female

- Educational level:

□School: □Year/Grade at School:

□ Bachelor’s degree □ Master’s degree □Name of the program:

- - Year of study at the University: first/second/third/fourth/fifth or trainee
- Name of school/university (if student):
- Phone number:
- Email:
- Nationality: ……………………………….

□ Emirati □ Arab (other than Emirati) □ Asian □ African

□Any other nationality (please specify) ……

- Current history of tobacco use/smoking:

□ Yes □No

- Current medical history (if applicable):

□ Diabetes □Hypertension □Dyslipidemia

□Hypothyroidism □ Polycystic ovary syndrome (for females)

□Any other (please specify) ……………….

- Current medication history (if applicable): …………………………
- Leg preferred to kick a ball

□ Right □Left □Both left and right

### The extended version of the Nordic Musculoskeletal questionnaire (NMQ-E)


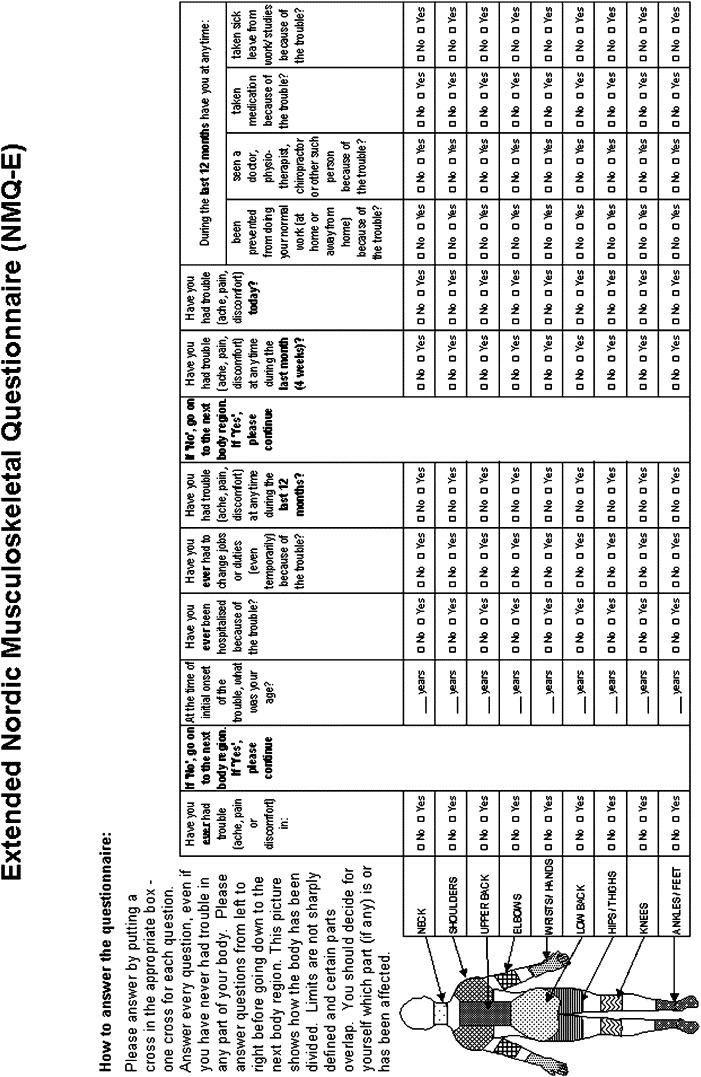


Reference: Dawson AP, Steele EJ, Hodges PW, Stewart S. Development and test-retest reliability of an extended version of the Nordic Musculoskeletal Questionnaire (NMQ-E): a screening instrument for musculoskeletal pain. J Pain. 2009;10(5):517–26.

**The Edinburgh Handedness Inventory - Short Form**

Please indicate with an “X” your preference in the use of hands in the following activities or objects:

| **Activity/ Object** | **Always left** | **Usually left** | **Both equally** | **Usually right** | **Always right** |
| --- | --- | --- | --- | --- | --- |
| **Writing** |  |  |  |  |  |
| **Throwing** |  |  |  |  |  |
| **Toothbrush** |  |  |  |  |  |
| **Spoon** |  |  |  |  |  |

Reference: Veale JF. Edinburgh handedness inventory–short form: a revised version based on confirmatory factor analysis. Laterality: Asymmetries of Body, Brain and Cognition. 2014;19(2):164–77.
